# Risk factors for multidrug-resistant tuberculosis among tuberculosis patients in Serbia: a case-control study

**DOI:** 10.1186/s12889-018-6021-5

**Published:** 2018-09-12

**Authors:** Maja Stosic, Dejana Vukovic, Dragan Babic, Gordana Antonijevic, Kristie L. Foley, Isidora Vujcic, Sandra Sipetic Grujicic

**Affiliations:** 1 Department of HIV/AIDS, STIs, Viral Hepatitis and TB, Public Health Institute of Serbia, “Dr Milan Jovanovic Batut”, Dr Subotica 5, Belgrade, 11000 Serbia; 2 Institute of Social Medicine, Faculty of Medicine, Belgrade University, Dr Subotica 9, Belgrade, 11000 Serbia; 3 Institute of Medical Statistics and Informatics, Faculty of Medicine, Belgrade University, Dr Subotica 9, Belgrade, 11000 Serbia; 4 Special Hospital for Lung Diseases, „Ozren”Ozrenska bb, Sokobanja, 18230 Serbia; 5 Department of Social Sciences and Health Policy, Division of Public Health Sciences, Wake Forest School of Medicine, Winston-Salem, North Carolina USA; 6 Institute of Epidemiology, Faculty of Medicine, Belgrade University, Visegradska 26, Belgrade, 11000 Serbia

**Keywords:** Tuberculosis, Multidrug –resistance, Risk factors, Case-control study

## Abstract

**Background:**

Multidrug resistant (MDR) tuberculosis (TB) represents TB which is simultaneous resistant to at least rifampicin (R) and isoniazid (H). Identifying inadequate therapy as the main cause of this form of the disease and explaining the factors leading to its occurrence, numerous social determinants that affect the risk of developing resistance are highlighted. The objectives of the study was to identify independent factors of MDR-TB among tuberculosis patients.

**Methods:**

Case-control study was conducted from 1st September 2009 to 1st June 2014 in 31 healthcare institutions in Serbia where MDR-TB and TB patients were treated. TB patients infected with MDR- *M. tuberculosis* and non MDR- *M. tuberculosis* strain were considered as cases and controls, respectively. Cases and controls were matched by the date of hospitalization. The data was collected using structured questionnaire with face to face interview. Bivariate and multivariable logistic regression analysis (MLRA) were used to identify determinants associated with MDR-TB.

**Results:**

A total of 124 respondents, 31 cases and 93 controls were participated in the study. MLRA identified six significant independent risk factors for the occurrence of MDR-TB as follows: monthly income of the family (Odds ratio (OR) = 3.71; 95% Confidence Interval (CI) = 1.22–11.28), defaulting from treatment (OR = 3.33; 95% CI = 1.14–9.09), stigma associated with TB (OR = 2.97; 95% CI = 1.18–7.45), subjective feeling of sadness (OR = 4.05; 95% CI = 1.69–9.70), use of sedatives (OR = 2.79; 95% CI = 1.02–7.65) and chronic obstructive pulmonary disease (OR = 4.51; 95% CI = 1.07–18.96).

**Conclusion:**

In order to reduce burden of drug resistance, strategies of controlling MDR-TB in Serbia should emphasize multi-sectorial actions, addressing health care and social needs of TB patients.

## Background

Multidrug-resistant (MDR) tuberculosis (TB) is TB in patients whose isolated bacilli are resistant in vitro to at least both anti-TB drugs (isoniazid and rifampicin). Identifying inadequate therapy as the main cause of this form of the disease and explaining the factors leading to its occurrence, the World Health Organization (WHO) highlights numerous social determinants that affect the risk of developing resistance [1]. Special attention was placed to poverty, poor living conditions, various causes of social vulnerability and reduced access to and availability of health services. In addition, the need for better understanding of the factors related to behavior (individual and community level) is stipulated [2]. Numerous articles document the connection between MDR-TB and social determinants of health: inadequate therapeutic regimen, improper dosages, inadequate drugs, short time of receiving treatment, poor quality of drugs as well as poor adherence to treatment regimen. Prison is often mentioned as a risk factor [2, 3], prolonged hospitalization [3–5], alcohol and the presence of HIV infection [6]. Social isolation is one of the most important determinants. The largest number of socially isolated patients start treatment too late and do not comply with the therapeutic regimen [7]. Stigma contributes to the isolation and its consequences, making it a significant determinant of MDR-TB [8]. A case control study in France, Germany, Italy and Spain found the following determinants of MDR-TB: intravenous drug use, belonging to migrants/asylum seekers population, income, institutional accommodation, previous pulmonary TB disease, imprisonment, contact with TB patient and immunosuppressive conditions other than HIV/AIDS, current TB and characteristics of the health service [9].

In Serbia the burden of TB has significantly decreased since 2005 due to TB Program implementation based on WHO Directly Observed Treatment (DOT), Short Course and STOP TB Strategy and financial support of Global fund to fight AIDS, tuberculosis and malaria (GFATM). It resulted in reduction of TB incidence by 58% in Serbia, reaching 13 per 100,000 population in 2014 [10]. However, there are concerns related to case management. There has been a decrease in treatment success rate of drug susceptible TB cases from 87% in 2009, 2010 and 2011 to 85%, 82% and 79% in 2012, 2013 and 2014, respectively. Treatment success rate of MDR-TB cases decreased from 71% in 2010 and 76% in 2011 to 57% in 2012 and 54% in 2013. The death rate and number of patients lost to follow up has increased [10].

In addition, TB remains a public health problem due to socio-economic crisis. There is a high unemployment rate in the country (18%) and at least 1 million people live below the poverty line [11]. Information on social determinants among MDR-TB and TB patients is limited in Serbia, with preliminary evidence suggesting that social determinants of health might be different in MDR-TB vs. drug susceptible TB patients.

The aim of the present study was to identify independent factors of MDR-TB among tuberculosis patients in order to provide evidence for planning of the targeted programmatic interventions to reduce burden of multidrug-resistant tuberculosis in Serbia.

## Methods

### Study design

Case control study was performed from 1st September 2009 till 1st June 2014 in 31 healthcare institutions in Serbia where MDR-TB and TB patients were treated. The study was approved by Ethic Committee of the Special Hospital for Lung Diseases Ozren – Sokobanja (No 2002/2014), the largest TB treatment facility and reference institution for hospital treatment of MDR TB in Serbia.

### Sample and procedure

The cases were consecutive MDR-TB patients of both sexes, aged 15 and older, diagnosed with multidrug-resistant TB for the first time at the time of the study. The controls were composed of persons diagnosed with drug susceptible TB for the first time during the study period. Three controls have been selected per each case. Cases and controls were matched by the date of hospitalization (the first three patients with TB who were hospitalized after diagnosing a patient with MDR-TB). Exclusion criteria included mental disability and inability to understand the objectives and procedures of the study.

In Serbia, all bacteriological confirmed TB patients are routinely tested for drug resistance. Definitive diagnosis of multidrug-resistant TB requires that *Mycobacterium tuberculosis* bacteria is detected as resistant to both isoniazid and rifampicin: isolating the bacteria by culture, identifying it as belonging to the *M. tuberculosis* complex and conducting drug susceptibility testing using solid or liquid media or by performing a molecular test to detect TB DNA and mutations associated with resistance [1].

The sample size was calculated using EPI-Info 3.02 statistical software. Parameters used to calculate the sample size were: prevalence of TB cases previously treated of 3.6% [12], odds ratio of MDR-TB for previously treated cases of 10.23 [13], a 1:3 ratio between cases and controls, 90% power and 95% confidence level. The result of 112 sample was increased 10% in case of non-response. Accordingly, the sample size was calculated to be 124 (31 cases and 93 controls). All cases and controls fulfilling the inclusion criteria were consecutively included in the study until the sample size was achieved.

### Measurements

We conducted an in-person survey of cases and controls, facilitated by health workers who were trained on survey administration prior to study initiation. The survey included 43 closed-ended questions and included the following eight groups of constructs: demographic and socio-economic data (gender, age, marital status, education, employment, type of settlement, household income); health behavior in the past 12 months (smoking, exposure to secondhand smoke, alcohol consumption, use of sedatives, tranquillizers, analgesics, substances); social support (number of people you can count on them when you have serious personal problems, possibility of being practically helped by neighbors if you are in need); knowledge of TB as a communicable disease and about predisposition for TB, attitudes (persistence of subjective feeling that people look them differently and that they will lose employment and friends due to TB as well as hiding the disease); mental health in the past 12 months (persistence of subjective feeling of nervousness, sadness, happiness and experience of stressful situations); exposure to harmful substances over the lifetime (vapor, gases); hospitalization over the lifetime; experience of being in a congregate setting (prison, migrant reception facility, home for elderly or disabled); disease specific factors (TB treatment interruptions and comorbidities) and help-seeking behavior. Some questions were used from “Health Survey in Serbia for 2013” (with the permission of the authorities) in order to ensure comparability of variables of the study participants and general population. Research instrument of “Health Survey in Serbia for 2013” were harmonized with the instruments of the European Health Survey second wave (EHIS wave 2) according to a defined internationally accepted indicators (ECHI, OMC, WHO, UNGASS, MDG) [14].

### Statistical analysis

Data were entered and analyzed using IBM SPSS Software V20.0 [15]. To obtain summary values for cases and controls separately we used descriptive statistical analyses. Bivariate analysis was performed to identify the crude association between dependent and independent variables. The dependent variable was presence of MDR-TB and the independent variables included socio-demographic, environmental, patient’s health and attitudes, health behavior and disease specific variables.

Statistical significance was determined using *p* < 0.05 as a cut-off point and odds ratio was used as a measure of the strength of association. Those variables which showed significant association (at *p* value ≤0.05) in bivariate analysis were collectively entered in a backward stepwise logistic regression procedure for multivariable logistic analyses, in order to assess the independent predictors of MDR-TB among the study participants. Variables which had shown statistically significant association during the bivariate analysis were: monthly income of the family, use of sedatives, use of tranquilizers, persistence of subjective feeling of nervousness, persistence of subjective feeling of sadness, experience of stressful situations in last 12 months, knowledge about predisposition of TB, social network, stigma, previous TB treatment history, default from treatment and chronic obstructive pulmonary disease (COPD).

## Results

A total of 124 respondents, 31 cases and 93 controls, participated in the study (Table 1). More than half of cases (58.1%) and controls (50.2%) belonged to the age group of ≤50 years. Males constituted 71.0% of the cases and 66.7% of controls. Currently not married respondents (61.3%) dominated among cases, while married (57%) among controls. Eight (25.8%) of cases and thirty three (35.5%) controls were unemployed. Almost half (47.6%) of cases and 15.4% of controls, had an estimated monthly income per family less than 100 Euro. There was no significant difference between these groups according to sex, age, education degree, occupation, marital status, residence and number of household members. Cases were significantly more likely to have incomes that were equal to or less than 100 Euros compared to controls.

**Table 1:** Bivariate analysis of socio-demographic characteristics of multidrug-resistant (MDR) and drug susceptible (DS) tuberculosis respondents

Variables	MDR-TB^a^ (*N* = 31)	DS-TB^b^ (*N* = 93)	OR (95% CI)	p value according to ULRA
	No (%)	No (%)		
Sex				
Male	22 (71.0)	62 (66.7)		
Female	9 (29.0)	31(33.3)	1.22 (0.50–2.97)	0.658
Age (years)				
≤50	18 (58.1)	47 (50.2)		
> 50	13 (41.9)	46 (49.5)	1.36 (0.59–3.10)	0.536
Education degree				
High school or lower	28 (90.3)	83 (89.2)		
University or higher	3 (9.7)	10 (10.8)	1.42 (0.81–2.49)	0.220
Occupation				
Unemployed	8 (25.8)	33 (35.5)		
Employed	23 (74.2)	60 (64.5)	0.72 (0.48–1.05)	0.091
Marital status				
Married	12 (38.7)	53 (57.0)		
Not married	19 (61.3)	40 (43.0)	0.48 (0.21–1.09)	0.081
Patient residence				
Rural	16 (51.6)	32 (35.2)		
Urban	15 (48.4)	59 (64.8)	1.97 (0.86–4.49)	0.108
Family size				
≤ 3	20 (64.5)	52 (55.9)		
≥ 4	11 (35.5)	41 (44.1)	1.43 (0.62–3.33)	0.402
Monthly income of the Family (Euro)				
≤100	10 (47.6)	12 (15.4)		
> 100	11 (52.4)	66 (84.6)	5.00 (1.74–14.35)	0.003

^a^Patients resistant to isoniazid and rifampicin (with or without resistance to additional anti-TB drugs); ^b^Patients infected with drug susceptible *M. tuberculosis* strain

There was no significant difference between the examined groups according to variables related to health behavior (Table 2). There was significant differences in mental health of the respondents. MDR-TB patients significantly more frequently used sedatives (Odds ratio (OR) =3.39; 95% Confidence Interval (CI) =1.32–8.67) and tranquilizers (OR = 3.89; 95% CI = 1.53–9.86) than TB patients. In addition, there was significant difference between the examined groups in persistence of subjective feeling of nervousness (OR = 3.34; 95% CI = 1.36–8.17), persistence of subjective feeling of sadness (OR = 3.88; 95% CI = 1.63–9.24) and experience of stressful situations in last 12 months (OR = 2.88; 95% CI = 1.16–7.09).

**Table 2:** Bivariate analysis of health behaviour and mental health of multidrug-resistant (MDR) and drug susceptible (DS) tuberculosis respondents

Variable	MDR-TB^a^ (*N* = 31) No (%)	DS-TB^b^ (*N* = 93) No (%)	OR (95% CI)	p value according to ULRA
Current smoker	15 (51.7)	36 (39.1)	1.67 (0.72–3.86)	0.233
Exposure to secondhand smoke	7 (22.6)	16 (17.2)	2.86 (0.84–9.75)	0.092
Exposure to harmful substances over the lifetime	16 (51.6)	46 (49.4)	1.09 (0.48–2.45)	0.836
Alcohol consumption	14 (45.1)	44 (47.3)	0.91 (0.40–2.07)	0.835
Use of sedatives	11 (35.4)	13 (13.9)	3.39 (1.32–8.67)	0.011
Use of tranquilizers	12 (38.7)	13 (13.9)	3.89 (1.53–9.86)	0.004
Use of analgesics	11 (35.4)	33 (35.4)	1.00 (0.43–2.34)	0.877
I consider myself nervous	13 (41.9)	16 (17.2)	3.34 (1.36–8.17)	0.008
Sometimes I feel so sad that nothing can cheer me up	19 (61.3)	28 (30.1)	3.88 (1.63–9.24)	0.002
Despite of disease I think I am happy?	18 (58.0)	73 (78.5)	0.76 (0.23–2.51)	0.654
Did something trouble you during the past 12 months?	23 (74.1)	46 (49.4)	2.88 (1.16–7.09)	0.022
Disease problems	10 (32.2)	16 (17.2)	1.53 (0.55–4.26)	0.408
Problems with the kids	4 (12.9)	5 (5.37)	1.73 (0.42–7.16)	0.452
Socio - economic conditions	9 (29.0)	13 (41.9)	1.73 (0.61–4.94)	0.307

^a^Patients resistant to isoniazid and rifampicin (with or without resistance to additional anti-TB drugs); ^b^Patients infected with drug susceptible *M. tuberculosis* strain

Also, MDR-TB patients significantly more likely reported difficulties obtaining help from neighbors when they are in need (OR = 0.41; 95% CI = 0.17–0.98) and had significantly higher levels of knowledge regarding predisposition of TB (OR = 2.00; 95% CI = 1.15–3.46) (Table 3) compared to TB patients. MDR-TB patients significantly more likely reported perceived stigma associated with TB (OR = 3.32; 95% CI = 1.40–7.86).

**Table 3:** Bivariate analysis of knowledge, social support and stigma of multidrug-resistant (MDR) and drug susceptible (DS) tuberculosis respondents

Variable	MDR-TB^a^ (N = 31) No (%)	DS-TB^b^ (N = 93) No (%)	OR (95% CI)	p value according to ULRA
TB is a communicable disease	29 (93.5)	81(87.0)	2.14 (0.45–10.18)	0.230
TB is inherited	12 (38.7)	21 (22.6)	2.00 (1.15–3.46)	0.013
Knowledge of tuberculosis^c^	11 (35.4)	19(20.4)	0.22(0.03–1.80)	0.160
There is at least one person who can help me if I have serious problems	2 (6.5)	9 (9.7)	0.64 (0.13–3.15)	0.587
It is easy to obtain help from neighbours when I am in need	17 (54.8)	70 (75.3)	0.41 (0.17–0.98)	0.044
People look at me differently because I have tuberculosis	24 (77.4)	60 (64.5)	1.88 (0.73–4.84)	0.187
I feel that I will lose friends and employment because I have tuberculosis(stigma)	21 (67.7)	36 (38.7)	3.32 (1.40–7.86)	0.006
I don’t like other people to know that I have tuberculosis	20 (64.5)	45 (48.3)	1.93 (0.83–4.49)	0.123

^a^Patients resistant to isoniazid and rifampicin (with or without resistance to additional anti-TB drugs); ^b^Patients infected with drug susceptible *M. tuberculosis* strain; ^c^Correct answer to the question whether tuberculosis is a communicable disease and whether it is inherited

Disease specific factors and co-morbidities of MDR-TB and drug susceptible TB respondents are presented in Table 4. MDR-TB patients significantly more likely reported previous TB treatment history (OR = 2.65; 95% CI = 1.14–6.16) and default from treatment (OR = 3.84; 95% CI = 1.41–11.11) than controls. Comorbidities were reported in more than one third of the respondents in both groups. Chronic obstructive pulmonary disease (COPD) was statistically significantly more common among MDR-TB patients (OR = 5.34; 95% CI = 1.39–20.40) than controls, as well as experience of hospital stay over a lifetime (OR =2.47; 95% CI = 1.08–5.69). However, there were no HIV positive respondents, there was no significant difference between the examined groups according to hypertension, diabetes, depression and anxiety, all co-morbidities, experience of being in a congregate setting over the lifetime and seeking help related to TB in public health care institution.

**Table 4:** Bivariate analysis of disease specific factors and co-morbidities of multidrug-resistant (MDR) and drug susceptible (DS) tuberculosis respondents

Variable	MDR-TB^a^ (N = 31) No (%)	DS-TB^b^ (N = 93) No (%)	OR (95% CI)	p value according to ULRA
Previous TB treatment history	16 (51.6)	28 (30.1)	2.65 (1.14–6.16)	0.023
Default from treatment	10 (32.2)	11 (11.8)	3.84 (1.41–11.11)	0.008
Hypertension	6 (19.4)	16 (17.2)	1.15 (0.40–3.27)	0.786
COPD^c^	6 (19.4)	4 (4.3)	5.34 (1.39–20.40)	0.014
Diabetes	3 (9.7)	10 (10.8)	0.89 (0.22–3.46)	0.866
Depression and anxiety	3 (9.7)	3 (3.22)	3.21 (0.61–16.83)	0.167
All co-morbidities^d^	12 (38.7)	27 (39.0)	0.65(0.28–1.52)	0.317
Experience of hospital stay over the lifetime	16 (51.6)	28 (30.1)	2.47 (1.08–5.69)	0.033
Experience of being in a congregate setting over the lifetime	6 (19.4)	12 (12.9)	0.62 (0.21–1.81)	0.381
Seeking help related to TB in public health care institution	29 (93.5)	80 (86.0)	2.35 (0.51–11.08)	0.278

^a^Patients resistant to isoniazid and rifampicin (with or without resistance to additional anti-TB drugs); ^b^Patients infected with drug susceptible *M. tuberculosis* strain; ^c^Chronic obstructive pulmonary disease; ^d^Hypertension, COPD, diabetes and depression

In multivariable logistic regression analysis, six variables were found to be significantly independent factors for the occurrence of MDR-TB such as: monthly income of the family (OR = 3.71; 95% CI = 1.22–11.28), defaulting from treatment (OR = 3.33; 95% CI = 1.14–9.09), stigma associated with TB (OR = 2.97; 95% CI = 1.18–7.45), persistence of subjective feeling of sadness(OR = 4.05; 95% CI = 1.69–9.70), use of sedatives (OR = 2.97; 95% CI = 1.18–7.45) and COPD (OR = 4.51; 95% CI = 1.07–18.96) (Table 5).

**Table 5:** Results of multivariable logistic regression analysis (dependent variable is multidrug-resistant tuberculosis respondents)

Variable	OR	95% Confidence interval		*p*-value
		Lower limit	Upper limit	
Monthly income of the family	3.71	1.22	11.28	0.021
Default from treatment	3.33	1.14	9.09	0.029
I feel that I will lose friends and employment because I have tuberculosis(stigma)	2.97	1.18	7.45	0.020
Sometimes I feel so sad that nothing can cheer me up	4.05	1.69	9.70	0.002
Use of sedatives	2.79	1.02	7.65	0.047
COPD^a^	4.51	1.07	18.96	0.040

^a^Chronic obstructive pulmonary disease

## Discussion

Although, Serbia is not a high MDR-TB burden country the study has provided baseline information about factors associated with MDR-TB which can support implementation of targeted interventions to decrease number of cases with MDR-TB.

Among socio-demographic factors, we identified that monthly income of the family ≤100 Euro is a risk factor for development of MDR-TB. It means that cases with lower income had almost four times higher risk of developing MDR-TB than controls. In almost 50% of cases, monthly income of the family of MDR TB cases was equal to level of poverty for Serbia, significantly lower than the corresponding general population [16]. These data are alarming given the circumstances that country does not allocate funds for social welfare payments for these patients. Although anti-TB drugs are available free-of-charge, TB patients still suffer from out-of-pocket payments with catastrophic consequences leading to poor treatment outcomes, disease spreading and possible development of drug resistance [17]. Siroka A et al., identified association between spending on social protection and TB burden [18]. Moreover, the results of the Nery et al. study in Brazil [19] provided evidence of a statistically significant association between the increase in cash transfer program coverage and a reduction in TB incidence rate, strongly suggesting the importance of the inclusion of TB patients in social protection programs during TB treatment. Harling G et al. study [20] documented association between treatment abandonment rates and low socioeconomic status as well, consequently reducing overall effectiveness of WHO STOP TB Strategy, despite the universal directly observed treatment (DOT) coverage in Brazil.

We found that the default from treatment is statistically significant independent risk factor associated with MDR-TB as confirmed by different studies in different countries [21–23]. It is one of the main barriers to TB control. It is defined as treatment interruption for at least two consecutive months [24]. Patients with non-adherence to treatment may remain infectious, experience increased risk of TB recurrence, TB-related mortality or increased probability of acquired drug resistance [25]. It is recognized as a common behavioral problem that initiated shifting from sanatorium care to community-based TB treatment [26]. Several studies documented risk factors for default associated with patients as alcoholism, substance abuse [27], unemployment, previous incarceration and homelessness [28].The consequences of treatment failure and further acquired drug resistance made directly observed treatment a high priority strategy for drug-resistant TB management. Directly observed treatment (DOT) is the most effective strategy worldwide for ensuring patients to take their medications correctly [29]. This level of care, however, requires increased workload and commitment than drug-susceptible disease and therefore need to be a shared responsibility of the society [29]. Proper use of incentives can improve treatment adherence as well. Food incentives together with social worker support improved treatment adherence in Singapore [30], Russia [31] and many other countries worldwide [32, 33]. Health system weaknesses are also identified as a factors contributing to poor MDR TB case management resulting in treatment default (absence of updated clinical guidelines, inappropriate or non-compliance with guidelines, poor training and supervision of health professionals, poor organization of TB program) [1]. Reduction of the duration of treatment for MDR-TB together with management of severe adverse reactions are also emphasized as the factors that can possible increase adherence to treatment [34]. Treatment of MDR TB requires prolonged use of multiple medications and most patients will experience difficulties in their tolerance. To avoid misunderstandings and defaulting, patients should be well informed about treatment regimens so that they can be recruited as partners who will invest in the success of the therapy together with the healthcare professional [35–38].

MDR-TB treatment in Serbia started with a Global Fund grant in 2009, and during the first 2 years treatment was given mainly for chronic and difficult-to-treat MDR-TB cases. Although many patients died during treatment, most (60%) were successfully treated in 2009; subsequently, they treatment success rate has been increasing (71% in 2010, 76% in 2011 and 77% in 2012). Despite good hospital capacity for MDR-TB patients in Serbia, measures are needed to improve ambulatory care services once patients with MDR-TB were discharged from hospital, with a focus on improving adherence to treatment [39]. After the end of Global fund support to TB program in Serbia, treatment success rate among MDR TB patients in Serbia decreased to 57% in 2013. Program is facing with high default rate. There are concerns related to case management, compliance with guidelines, capacity building, supervision and patient centered care and support [39].

Also, our study showed that MDR-TB cases are almost three times more frequently experienced stigma associated with TB, used sedatives and felt very sad compared to controls. TB is known as a highly stigmatized disease. MDR-TB patients are particularly affected having in mind long (20 month) and arduous treatment, regime consisted of powerful anti-TB drugs with severe side-effects that can affect mental health, higher case fatality and lower cure rates [1].Many studies identified stigma associated with a negative treatment outcome since it is obstacle in cooperation and communication with the health service and often results by delays in seeking medical help, diagnosis and treatment, contributes to treatment interruptions, treatment failure, recurrence and resistance [40, 41]. Our study data, together with findings of the other authors, highly suggest that mental health services have to be incorporated in TB control programs to support adherence to treatment of the patients and to reduce the influence of emotional distress and iatrogenic mental disorders during treatment, since those conditions are not routinely addressed by TB specialists [42, 43].

Furthermore, we found chronic obstructive pulmonary disease as a factor associated with MDR TB. Study of Zhao JN et al. [13] also indicated that pulmonary TB patients complicated with COPD had a higher chance of developing MDR-TB. COPD and TB have common risk factors such as smoking, low socioeconomic status and dysregulation of host defense functions resulting in synergistic damage of the lungs, with interaction on immunologic and cellular level leading to reduce treatment efficacy, persistent infectivity and relapse (indicator of reduced treatment effectiveness) [44]. COPD patients often suffer from conditions such impaired muco-ciliary clearance, long-term inhaled corticosteroids therapy, increasing potential possibility of TB infection and active disease. Studies from Sweden and China identified that COPD patients with severe airflow obstruction have persistent airway inflammation and decreased mucosal defense function, predisposing to increase the infection by drug resistant strains [45, 46].

This study contributed to increasing overall knowledge and follow up studies can be conducted to understand the further associations. However, this study did not indicate that sex, education level, alcoholism, previous TB treatment and HIV infection were significantly associated with development of MDR-TB, as several other studies [21, 37, 38, 13, 44].

The major strength of this study was inclusion of all MDR-TB patients living in all parts of Serbia and selecting the cases and controls based on the result of molecular technique, line probe assay. Some increase of sensitivity of the case-control study was achieved by increasing the number of controls (three controls were used).

Several limitations of this study should be considered in the interpretation of the results. First, we obtained information about the year before participants became MDR-TB and TB cases, and therefore, recall biases were unavoidable and potentially affected the results. Second, some information, such as annual per capita income and use of different substances, might be inaccurate, because it was collected by self-reporting.

Despite these limitations, the risk factors associated with MDR-TB provided important information for health policy and planning of targeted programmatic interventions for future improvements of MDR TB prevention and control in Serbia. To the best of our knowledge, the present case-control study is the first investigation of risk factors for drug -resistant TB in the Serbia.

## Conclusions

In light of study findings, strategies of controlling MDR-TB in Serbia should emphasize on inter-sectorial actions to improve living conditions and reduce the high rates of poverty of the patients by their involvement in programs of social support (material and psychological), retraining programs, and programs of social entrepreneurship. To achieve better adherence to TB treatment and reduce defaulting from treatment MDR-TB case management has to be improved (clinical guidelines updated, continuous training programs, supervision and support of health professionals on service delivery level provided) and support to patients and their families provided during treatment. To reduce stigma associated with TB and bad mental health of the MDR-TB patients, it is necessary to include mental health services into the TB care process.

Finally, having in mind immigration as a potential treat to TB control, protocols for TB case detection among migrants has to be developed whose origin are from MDR-TB strain dominant countries.

## Abbreviations

AIDS: Tuberculosis and malaria
COPD: Chronic obstructive pulmonary disease
GFATM: Global fund to fight
HIV: The human immunodeficiency virus
I: Isoniazid
MDR: Multidrug resistant
MDR-TB: Multidrug resistant tuberculosis
R: Rifampicin
TB: Tuberculosis
WHO: World Health Organization
